# Reflection of Dietary Iodine in the 24 h Urinary Iodine Concentration, Serum Iodine and Thyroglobulin as Biomarkers of Iodine Status: A Pilot Study

**DOI:** 10.3390/nu13082520

**Published:** 2021-07-23

**Authors:** Katelyn Hlucny, Brenda M. Alexander, Ken Gerow, D. Enette Larson-Meyer

**Affiliations:** 1 Department of Family and Consumer Sciences, University of Wyoming, Laramie, WY 82071, USA; katielangaas@gmail.com; 2 Department of Animal Science, University of Wyoming, Laramie, WY 82071, USA; balex@uwyo.edu; 3 Department of Mathematics and Statistics, University of Wyoming, Laramie, WY 82071, USA; gerow@uwyo.edu; 4 Department of Human Nutrition, Foods and Exercise, Virginia Tech, Blacksburg, VA 24061, USA

**Keywords:** iodine status, biomarkers, validation, nutritional exposure, dietary biomarkers, iodine intake, urinary iodine concentration, serum iodine, thyroglobulin, food frequency questionnaire, dairy products

## Abstract

Background: The iodine status of the US population is considered adequate, but subpopulations remain at risk for iodine deficiency and a biomarker of individual iodine status has yet to be determined. The purpose of this study was to determine whether a 3 day titration diet, providing known quantities of iodized salt, is reflected in 24 h urinary iodine concentration (UIC), serum iodine, and thyroglobulin (Tg). Methods: A total of 10 participants (31.3 ± 4.0 years, 76.1 ± 6.3 kg) completed three, 3 day iodine titration diets (minimal iodine, US RDA, (United States Recommended Daily Allowance), and 3× RDA). The 24 h UIC, serum iodine, and Tg were measured following each diet. The 24 h UIC and an iodine-specific food frequency questionnaire (FFQ) were completed at baseline. Results: UIC increased an average of 19.3 μg/L for every gram of iodized salt consumed and was different from minimal to RDA (*p* = 0.001) and RDA to 3× RDA diets (*p* = 0.04). Serum iodine was different from RDA to 3× RDA (*p* = 0.006) whereas Tg was not responsive to diet. Baseline UIC was associated with iodine intake from milk (r = 0.688, *p* = 0.028) and fish/seafood (r = 0.646, *p* = 0.043). Conclusion: These results suggest that 24 h UIC and serum iodine may be reflective of individual iodine status and may serve as biomarkers of iodine status.

## 1. Introduction

Iodine is an essential, rate-limiting element for the synthesis of thyroid hormones, which is currently the only known physiological role of iodine. Ensuring adequate iodine intake is important for all adults, but particularly important for women of childbearing age. Inadequate intake and deficiency during pregnancy can influence brain, nerve and muscle development in the growing fetus and result in growth and developmental abnormalities [1].

Iodine status in the US is generally thought to be adequate [2,3]. However, select populations are susceptible to insufficient iodine status due to geographical location, food intake practices, or increased iodine needs (e.g., pregnancy and lactation). Subpopulations at increased risk for low iodine intake include pregnant and lactating women [4,5,6], vegans [7,8,9] and vegetarians [7,8,9,10,11], those who avoid seafood and/or dairy [12], follow a sodium restricted diet [13,14], or eat local foods in regions with iodine-depleted soils [1,15]. The Institute of Medicine, American Heart Association, and the 2020–2025 Dietary Guidelines for Americans [16] have advocated for decreasing sodium intake to less than 2300 mg/day [17] and more prudently to less than 1500 mg/day [18], which could be reducing Americans’ intake of iodized salt. Additionally, trends toward local and plant-based diets may negatively influence iodine status depending on food selection and dietary intake patterns (e.g., avoidance of seafood, dairy and eggs) and the content of local soils. Therefore, despite presumed adequate iodine status in the general US population, certain dietary choices may directly influence iodine status and hence, influence thyroid function. Additionally, dietary patterns low in iodine content are of particular concern for women of reproductive age and pregnant and lactating women due iodine’s importance during fetal growth and development. 

The 24 h urinary iodine concentration (UIC) is considered the gold standard for assessing iodine status in populations, as approximately 90% of dietary iodine is excreted in the urine of healthy and iodine replete adults [19]. However, 24 h urine collections are cumbersome for the patient, prone to collection and methodological errors, expensive to measure by state-of-the art procedures employed by most clinical laboratories (i.e., mass spectroscopy) [20], and may not adequately reflect iodine status in individuals [21]. For example, 24 h UIC may better represent acute (e.g., days) versus chronic iodine status [22] because of variations in 24 h iodine excretion due to within-day hydration status changes [23] and other unknown variables [22]. While other biomarkers for assessment of status have been suggested, including 24 h total urinary iodine excretion (UIE) [19,24], serum iodine [25] and thyroglobulin (Tg) concentration [26], use of these markers (as well as 24 h UIC) must be better validated in healthy subjects against known quantities of iodine intake. 

The primary purpose of this pilot study was to determine whether a 3-day titration diet (which provided known quantities of iodized salt) is reflected in 24 h UIC, UIE, serum iodine, thyroglobulin biomarkers. Secondary purposes were to evaluate the association between baseline 24 h UIC and habitual iodine intake and observe the contribution of iodine-containing foods to total iodine intake assessed by an iodine-specific food frequency questionnaire (FFQ). We hypothesized that 24 h UIC, UIE and serum iodine would increase as iodized salt consumption increased and that Tg would increase when the iodine intake exceeds the iodine Recommended Daily Allowance (RDA) of 150 ug. We also hypothesized that baseline 24 h UIC would be associated with the intake frequency of dairy, eggs, and iodized salt and that these same food items would be associated with the total iodine intake as estimated by the FFQ.

## 2. Materials and Methods

**Figure 1:**
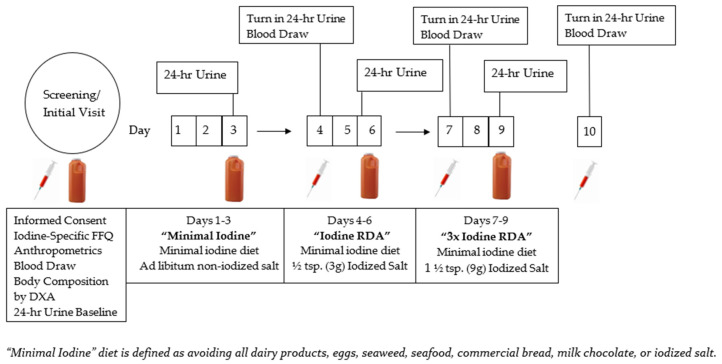
Protocol schematic showing the iodine titration diet progression at the initial visit and ending on day 10. The “syringe” graphic indicates scheduled blood draws and “orange urine collection jugs” indicate 24 h urine collections time points. A minimal iodine diet is defined at the bottom of the schematic.

This pilot study was conducted between February and June of 2020 with participants completing a 9 day study period in February and March 2020. The study schematic is shown in Figure 1. All research procedures were reviewed and approved by the Institutional Review Board of the University of Wyoming. This study evaluated the responsiveness of a three biomarkers of iodine status using three known quantities of dietary iodine consumption. Participants were notified of any possible risks prior to giving written formal consent to participate in this study. Volunteers were recruited through flyers and word of mouth from the University of Wyoming campus and the local community.

### 2.1. Overview of Screening and Baseline Testing

In early February, interested volunteers completed a screening interview to ensure preliminary eligibility. Interested and eligible volunteers reported to the lab between 8 and 9 am for an initial/baseline visit, which involved provision of written informed consent, completion of a health history questionnaire, and an iodine-specific FFQ. Blood was drawn for analysis of a complete blood count (CBC), thyroid stimulating hormone (TSH) and thyroglobulin antibodies, and was also used for analysis of baseline serum Tg and iodine concentrations. The 24 h urine was collected for analysis of baseline urinary iodine concentration (UIC) as outlined in Figure 1. Body composition was assessed by dual energy x-ray absorptiometry (DXA (Lunar Prodigy, GE Healthcare, Farfield, CT, USA)) during a separate visit scheduled within 10 days of baseline. Following the baseline visit, participants completed three, 3 day titration diets with increasing concentrations of iodine from iodized table salt (Morton Salt, processed 10/5/19, Providence, Rhode Island, US). These diets included “minimal iodine”, iodine close to the RDA (“RDA”) and iodine close to three times the RDA (“3× RDA”) as explained below (see Section 2.5). The 24 h UIC was measured on the last full day of each diet intervention and is explained in detail below (see Section 2.6 24 h Urine Collections).

### 2.2. Participants

Participants included 5 women and 5 men, aged 19–56 years with a BMI (kg/m^2^) ≥ 18 to ≤40. A sample size of *n* = 10 was selected for this pilot study due to resource constraints. Individuals were screened by phone and deemed ineligible if they answered “yes” to any or all questions regarding presence of thyroid disorders, currently pregnant or lactating, current use of commercial douches (which may contain iodine), presence of autoimmune disease history/status, and current use of tobacco (including smoking) and medications. Additional exclusion criteria include not willing to comply with the salt/iodine restricted aspects of this study, not being available to/agreeing to fully participate in all aspects of this study and oblige to all restraints during the iodine-controlled 9 days of this study (including avoidance of strenuous exercise; due to potential iodine loss via sweating [27,28], having extreme dietary habits that would result in exceptionally low or high iodine intake, currently taking or have taken iodine or kelp supplements or medications containing or interfering with iodine/thyroid status in the past month, having a history of thyroid disorders, Addison’s disease, or Cushing’s syndrome, a history of major chronic diseases, having laboratory signs or symptom’s suggestive of anemia and/or hypo/hyperthyroidism. Results of the CBC, TSH, thyroglobulin antibodies and health history were used to ensure eligibility to proceed to the next steps of the study protocol.

### 2.3. Baseline Measurements and Testing

Basic anthropometric testing, which included height, weight and body composition measurements, was performed for descriptive proposes. Height and weight were measured in minimal clothing using a stadiometer (Invicta Plastics, Leicester, UK) to the nearest 0.1 cm and a standing digital scale (Tanita, Tokyo, Japan) to the nearest 0.1 kg. Body composition was measured by DXA. A pregnancy test using a urine test strip was completed immediately before the DXA to ensure female participants were not pregnant. Screening blood work (CBC, TSH, thyroglobulin antibodies), Tg and 24 h UIC were measured by a commercial laboratory as detailed in Section 2.7 below. 

### 2.4. Baseline Iodine Intake and Frequency of Intake of Iodine-Containing Foods

The iodine-specific FFQ (Appendix A) evaluated the frequency of consumption of 36 food items, over the past month, known to have significant iodine content (e.g., seafood, seaweed, dairy, egg). Our FFQ is a modified version of a previously validated FFQ by Gostas et al. [29], which found estimated total iodine intake from the FFQ to be correlated with 24 h UIC in over 100 healthy female and male participants. Our modified FFQ omitted six low-iodine-containing foods that were not of interest to our study and asked additional questions about habitual iodized salt intake. Frequency was evaluated according to the following responses: (a) never or less than one time per month; (b) one to three times per month; (c) one time per week; (d) two to four times per week; (e) five to six times per week; (f) one time per day; (g) two to three times per day; (h) four to five times per day; (i) or six or more times per day. Daily intake of iodine was estimated by multiplying the frequency midpoint by the average content of each iodine-containing food and expressed as ug/day (assuming 30 days per month), as previously outlined by Halliday et al. [30]. As iodine content is not available in food composition tables or databases, iodine content of the food items in the FFQ was derived from several sources [29] with most data from the ongoing Total Diet Study (TDS). Iodine content in the TDS is listed per 100 g of the selected food item. Iodine content was recalculated from the TDS to iodine per serving size, to match the household measured listed in the FFQ. The iodine content in other sources was also converted to adjust given units to iodine per serving size. 

### 2.5. Iodine Titration Diet and Biomarker Protocol

Following the baseline urine collection, volunteers then completed three, 3 day iodine titration diet periods as shown in Figure 1. 

Experimental treatments were designed to provide minimal iodine, close to the recommended dietary allowance (RDA), and 3× the RDA. The “Minimal Iodine” diet was defined as avoiding all dairy products, eggs, seaweed, seafood, commercial bread, milk chocolate, and iodized salt and was completed on study days 1–9. Ad libitum quantities of non-iodized salt was provided for participants on only the “Minimal Iodine” diet. The participants received 3.0 g (~1/2 tsp) of iodized salt/day on the “RDA” diet which took place on study days 4–6. On study days 7–9, participants received 9.0 g (1 ½ tsp) of iodized salt/day on the “3× RDA” diet. Participants were instructed to completely consume the premeasured quantities of iodized salt on study days 4–9 in any manner they chose (sprinkled on food, dissolved in a drinkable solution, etc.) and return empty containers on day 10 (the end of this study). Blood was drawn for analysis of concentrations of serum iodine and Tg in the morning between 8 and 9 am on days 4, 7, and 10. Returning the empty containers and verbal communication served as confirmation that the participants completely consumed the provided iodized salt.

### 2.6. The 24 h Urine Collections

Volunteers completed a total of four 24 h urine collections. Participants were instructed to void and discard the first morning urine sample and then collect all subsequent samples for 24 h ending with the first sample upon waking the next day. Females were provided urine collector pans which were placed under the toilet seat during urination to allow for ease of collection and pouring of urine into the 24 h collection container. Refer to Figure 1, which indicates 24 h urine collections were completed on study days 3, 6, and 9, in addition to baseline. Total urine volume was measured at minimal, RDA, and 3× RDA time points using a 2 L graduated cylinder.

### 2.7. Analysis of Iodine Status Biomarkers in Urine and Blood

Serum iodine and 24 h UIC were measured by a local laboratory (Region West, Scottsbluff, NE, USA) with analysis completed by a contract national laboratory (Mayo Clinic, Rochester, MN, USA). Serum Tg was analyzed at the University of Wyoming using a Human Tg ELISA (Enzyme-Linked Immunosorbent Assay) kit. Specifically, 24 h UIC and serum iodine were measured using Inductively Coupled Plasma-Mass Spectrometry and serum Tg concentration was measured by in vitro enzyme-linked immunosorbent assay. The 24 h UIE was calculated by multiplying 24 h UIC by total urine volume.

### 2.8. Post-Study Questionnaire

Participants were provided with a post-study questionnaire on day 10, following the last 24 h urine collection. Participants were asked to rate difficulty of consuming iodized salt on the RDA and 3× RDA diets and collecting urine at each of the 24 h urine collections on a Likert scale of 1–5, with 1 being the easiest and 5 being the most difficult. Additionally, participants were asked open-ended questions that included the most difficult part of completing this study, how the provided iodized salt was consumed, and whether normal eating patterns changed to accompany the added salt. Participants were asked whether all provided salt was consumed on the specified day and whether all urine was collected during each 24 h collection. 

### 2.9. Statistical Analysis

Data were analyzed using Minitab statistics software (Minitab LLC, State College, PA, USA; version 19.1). Response Feature Analysis (RFA) [31] was used to compare differences in UIC and serum iodine values but not Tg due to lack of difference at data collection points. Linear regressions were fitted to UIC and serum iodine measures for each individual participant. A paired t-test was used to compare 24 h UIC, serum iodine, and Tg values at minimal and RDA values and the same biomarkers were compared at RDA to 3× RDA. Multi-comparisons for each sex were not performed due to small sample size. Correlation coefficients (Spearman Rank) were used to evaluate the associations between total daily iodine intake and baseline 24 h UIC and iodine intake from specific foods (dairy, eggs, seafood, seaweed, iodized salt, etc.). Data are expressed as the means ± SEM unless otherwise specified. Significance was set at *p* < 0.05.

## 3. Results

### 3.1. Participant Characteristics 

The physical characteristics and baseline biochemical data of the five male and five female participants are summarized in Table 1. Two participants reported following a vegetarian diet and eight were omnivores. Eleven participants were recruited. However, one female dropped out of this study following the screening visit due to illness. Data for this participant are not included. Most of the participants were of normal BMI (18.5–24.9), with two men in the overweight category (25–29.9) and one female in the obese category (≥30). Thyroglobulin antibodies were <1.8 IU/mL (lowest detectable range) for all participants, indicating a very low possibility that any participant had an autoimmune thyroid disorder which could interfere with use of Tg as a marker of iodine status [32]. None of the participants smoked. All participants reported having restrained from strenuous physical activity causing excessive sweating for the duration of this study.

### 3.2. Baseline Daily Iodine Intake and Frequency of Intake of Iodine-Containing Foods

No participants reported having consumed supplements containing iodine. Daily iodine intake averaged 265.6 ± 28.2 ug (median: 264.5 ug; range: 93.8 to 401.7 ug) and was not different by sex (*p* = 0.55). The mean and median iodine intake was higher than the US RDA for male and non-pregnant female adults of 150 µg/day; however, 10% (*n* = 1) had estimated intakes less than the RDA and no participants had an intake greater than the Tolerable Upper Limit of 1100 ug/day. The estimated average daily iodine intake from contributing foods are as follows: total dairy (186.5 ± 36.9) (milk, yogurt, cheese, cottage cheese), milk (126.3 ± 34.1), eggs (27.3 ± 12.1), total fish and seafood (4.1 ± 1.3), seaweed (1.6 ± 0.5), and iodized table salt (42.8 ± 15.8). Total estimated iodine intake from the FFQ was associated with reported total dairy (r = 0.830, *p* = 0.003) and milk intake (r = 0.688, *p* = 0.03), but not with egg (r = 0.101, *p* = 0.78), fish and seafood (r = 0.457, *p* = 0.18) seaweed (r = 0.503, *p* = 0.14), or iodized salt intake (r = −0.235, *p* = 0.51). 

### 3.3. Baseline 24 h UIC and Iodine Status

**Figure 2:**
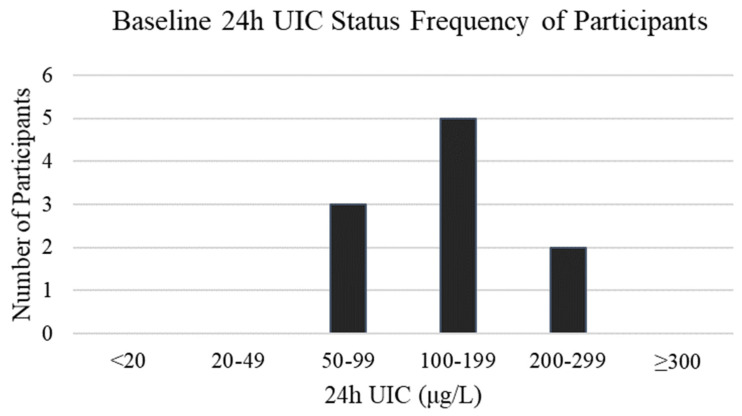
Iodine status based on the World Health Organization (WHO) criteria for urinary iodine concentration (UIC) [13]: <20 µg/L (Severe Deficiency); 20–49 µg/L (Moderate Deficiency); 50–99 µg/L (Mild Deficiency); 100–199 µg/L (Adequate); 200–299 µg/L (Above Adequate); ≥300 µg/L (Excessive).

Baseline 24 h UIC ranged between 67 and 253 µg/L. The average value was 135 µg/L and the median value was 121 µg/L. The frequency of the categories of iodine status based on World Health Organization (WHO) criteria is shown in Figure 2. Average baseline serum Tg fell within the normal range (≤33 ng/mL) with 1 participant having a Tg value > 33 ng/mL, indicating compromised status. 

### 3.4. Relationship between Baseline Iodine Intake and UIC

Estimated total iodine intake from the FFQ was not correlated with UIC (r = 0.273, *p*= 0.446). FFQ-estimated iodine intake was not correlated with predicted iodine intake using the equation of Zimmerman UIC (r = 0.248, *p* = 0.489) which incorporates UIC and body mass. UIC, however, was associated with milk (r = 0.688, *p* = 0.028) and fish/seafood intake (r = 0.646, *p* = 0.043), but not with reported total dairy intake (r = 0.515, *p* = 0.128), egg consumption (r = −0.241, *p* = 0.503), seaweed consumption (r = −0.121, *p* = 0.740), or iodized table salt use (r = −0.512, *p* = 0.130).

### 3.5. Effect of Titration Diet on Iodine Status Biomarkers

Titration diet effects on 24 h UIC, serum iodine and Tg are shown in Table 2. 

Urine volumes varied widely among participants (900–4150 mL), and averaged 2155 ± 195 mL, 1972 ± 210 mL and 2200 ± 290 on the minimal, RDA and 3× RDA diets, respectively. Both UIC and UIE increased from minimal to RDA (*p* < 0.001 for both) and RDA to 3× RDA (*p* = 0.04 and *p* = 0.002, respectively). The average UIC intercepts and slopes from minimal iodine to 3× RDA produced a regression as follows: UIC = 39.5 + 19.3 (grams iodized salt). Thus, UIC increased an average of 19.3 μg/L for every gram of iodized salt consumed. Regression line slopes and intercepts were not different between sexes (*p* = 0.27 and *p* = 0.46, respectively). 

Serum iodine did not increase from minimal to RDA (*p* = 0.67) but increased significantly from RDA to 3× RDA (*p* = 0.006). The average serum iodine intercepts and slopes from minimal iodine to 3× RDA produced a regression as follows: serum iodine = 61.2 (± 2.0) + 0.6 (± 0.2) grams iodized salt. Serum Tg concentration, however, was not different from minimal iodine to RDA (*p* = 0.94) or from RDA to 3× RDA (*p* = 0.68).

### 3.6. Post-Study Questionnaire Responses

Nine of ten participants reported consuming all the provided iodized salt on the RDA and 3× RDA diets. One participant reported consuming 75% of provided iodized salt on day 9 (3× RDA diet). Participants reported adding the iodized salt to reduced or unsalted homemade meals, margaritas, caramel, and 7 of the 10 reported dissolving the salt in water to drink.

Seven of 10 participants reported collecting 100% of urine output at each 24 h collection. One female participant reported missing 100–200 mL during the fourth 24 h urine collection (3× RDA diet). Another female missed a single urine collection during the 24 h collection period, and one male reported occasionally forgetting to collect a urine. 

Overall, participants reported the most difficult part of this study was avoiding eggs, dairy, and commercial bread, remembering to collect all urine, and consuming the 9 g of iodized salt on 3× RDA diet. All ten participants rated consuming all salt on RDA diet and collecting all urine as a 1, 2, or 3 difficulty on a 1–5 Likert scale of difficulty with 5 being the most difficult. Five of ten participants rated consuming all salt on 3× RDA diet as a 4 or 5 difficulty rating. Participants reported changing their normal eating pattern to accommodate additional salt by eating lower sodium foods and adjusting sodium content in homemade recipes, eating larger portions to make food more palatable, and increasing frequency of meals. They also reported their normal eating pattern changed by avoiding dairy, eggs, and bread.

## 4. Discussion

The primary purpose of this pilot study was to determine whether a titration diet (which used known quantities of iodized salt) was reflected in the 24 h UIC, serum iodine, and thyroglobulin biomarkers of iodine status. Secondary purposes were to evaluate the association between baseline 24 h UIC and habitual iodine intake and observe the contribution of iodine-containing foods to total iodine intake assessed by an iodine-specific FFQ. Overall, in our sample of 10 participants, we found that 24 h UIC measures (including 24 h UIE) were increased as iodized salt consumption increased. Serum iodine, on the other hand, did not increase from the minimal iodine diet to the iodine RDA diet but was elevated when three times the RDA was consumed. In contrast, serum thyroglobulin concentrations were not different during the titration diet period. As estimated by the iodine-specific FFQ, only milk and total dairy intake were associated with estimated total iodine intake, whereas only milk and fish/seafood intake was associated with 24 h UIC.

To the best of our knowledge, this is the first study to perform a controlled titrated iodine diet using iodized salt as an iodine source to evaluate the effects on iodine status biomarkers. A previous study providing oral iodine capsules (225μg/day of potassium iodide) to pregnant participants during the first trimester found that the urinary iodine/creatinine ratio increased from a median of 53 μg/g to 150–249 μg/g by the third trimester [33]. Women consumed their typical diet during the supplemental period. Additional published studies have found increases in UIC and presumed iodine status in children and adults from iodine deficient areas following initiation of a salt iodization program [34] or oral iodine supplementation in the form of capsules [33,35] or poppyseed oil [36,37], and by a single-dose injection of intramuscular iodized oil [38]. In our study, the use of iodized salt was a practical way to consume iodine and represented a real-life addition of iodine to the diet (due to iodination of table salt in many countries). An additional strength of this study was that we simultaneously evaluated 24 h UIC along with serum iodine, Tg, and UIE. The 24 h UIC, which is the “gold standard” to assess iodine status in populations and currently, may be the most representative of individual iodine status.

The primary purpose of this study was to explore use of potential biomarkers of individual iodine status including UIC, and serum iodine and Tg concentrations. Although 24 h UIC is considered a population iodine status marker, our results suggest that of the iodine status biomarkers evaluated, 24 h UIC has promise as an individual biomarker in that it is sensitive to changes in short term dietary intake. According to our regression model (from minimal iodine intake up to 9 g of iodized salt), 24 h UIC increased an average of 19.3 μg/L for every gram of iodized salt consumed. A gram of iodized salt contains 45 μg of iodine. Thus, an increase of 45 μg of dietary iodine increased the 24 h UIC by an average of 19.3 μg/L in our sample. As the 24 h UIC was completed on the third day of each diet, these results demonstrate that the 24 h UIC was sensitive to recent changes in iodine intake. The same increase in 24 h UIC was observed in the 24 h UIE. The advantage of total UIE is that it adjusts for urine volume and hydration status that can vary among individuals and throughout the day. For example, in our study, we had one participant with daily total urine volumes of 900 mL and another with a daily volumes of more than 4 L. Measuring urine volume to calculate 24 h UIE may be useful when evaluating iodine status of pregnant and lactating women to ensure this population is increasing water intake to the recommended amount (3 L for pregnant women and 3.8 L for lactating women). Since iodine stored in the thyroid is unaffected by changes in short-term iodine intake in iodine-sufficient individuals (i.e., thyroid stores are sufficient), urinary iodine excretion is primarily representative of recent iodine intake. If thyroid stores were not sufficient, more dietary iodine would likely be taken up by the thyroid (to help replete stores) and less would be excreted in urine [39,40]. This concept was demonstrated in our study. As iodine intake increased, the average urinary iodine excretion increased indicating that as intake of iodine exceeds needs and thyroid stores, more iodine will be excreted in the urine. Anderson et al. found that greater than ten spot urines or greater than seven 24 h UICs are required to estimate an individual’s iodine status in free living individuals within a precision range of ±20% [41]. Individuals in this study presumably had variable daily intake of iodine which were not simultaneously estimated during this study. The amount of iodine uptake by the thyroid, and the subsequent amount of iodine excreted in the urine, depends on the typical iodine intake of the individual [42]. If daily iodine intake is >50 ug, the uptake of iodine by the thyroid is maintained, but if daily iodine intake is <50 ug the thyroid’s iodine stores become depleted [29]. Wainwright and Cook suggested that multiple 24 h UICs be collected over a prolonged period of time to provide the best representation of usual iodine intake and to account for seasonal [43], circadian rhythm [44], and iodine and hydration status variations [21]. However, collection of multiple 24 h UICs would be even more of a burden to the patient than a single collection and be subject to collection error including missed urine samples. The difficulty of collecting 24 h urines and additional limitations [25,29,45,46,47,48,49] that include being cumbersome to the patient and being subject to within-day hydration and iodine intake changes prompted us to evaluate other potential biomarkers of iodine status (serum iodine and Tg) in the current study. 

Our finding that serum iodine concentrations were not different from minimal iodine to RDA but were increased from RDA to three times the RDA provides some support that serum iodine may be a potential alternative marker of iodine status. We initially hypothesized that serum iodine concentration would increase throughout the titration diet as iodine intake increased. Although research is limited on the use of serum iodine as an iodine status biomarker, previous research from mostly epidemiological studies in Chinese populations, have concluded that serum iodine may be more indicative of long-term (or typical) iodine status rather than recent iodine intake because serum iodine was observed to differ among location of residence but not between sexes or age groups, or according to smoking or exercise status [25,45]. The difference between our findings and those previously reported may be due to differences in study period duration. Studies conducted in Chinese populations with varying iodine status found that when iodine in the external environment increased (salt fortification and increased iodine in water supply), UIC increased while serum iodine remained more stable in comparison [25,45]. Another study conducted in Chinese adults found serum iodine was positively correlated with total T3 and T4 and free T4 concentrations [46], suggesting that serum iodine appears to most closely represent the bioavailable iodine for the thyroid gland, with 80–90% of total serum iodine incorporated into thyroid hormones [46,50]. While in overall agreement with previous studies, our study found significant increases in serum iodine only when iodine intake is excessive. This may be because our participants were presumably iodine replete at the start of this study and/or that the 3 day duration of this study was not of sufficient duration for serum thyroid to fall due to minimal iodine intake. Thus, more long-term studies (over weeks or months instead of days) may be needed to evaluate long-term serum iodine concentration changes.

In contrast to our hypothesis, serum thyroglobulin concentrations were not different as iodized salt consumption increased from minimal iodine (~0 g iodine) intake to 9 g iodized salt (45 μg of iodine)/day. Like serum iodine, this observation may be due to thyroglobulin’s lack of sensitivity to recent changes in iodine intake. Several previous cross-sectional studies [51,52,53] collectively reported inverse associations between serum Tg concentration and iodine status (determined by spot UIC and spot urinary iodine excretion (UIE)) within iodine deficient and excessive populations across the globe [51,52,53]. Because the studies determined iodine status based on a single-spot UIC or UIE sample, individual iodine status could not be determined [53]. Following a salt iodization program, Vejbjerg et al. found median serum Tg decreased from 10.9 to 8.7 mg/L (*p* < 0.001) in an area with previously mild iodine deficiency and decreased from 14.6 to 8.9 mg/L (*p* < 0.001) in an area with previous moderate iodine deficiency. Decreases in serum Tg are reflective of better iodine status because when iodine intake is insufficient, low circulating levels of T4 stimulate the synthesis and release of thyrotropin-releasing hormone, which subsequently increases the production of TSH; this then stimulates Tg synthesis, causing an increase in serum Tg [26]. Even though serum TSH increases during iodine deficiency, TSH concentrations often remain within normal reference ranges and thus is not considered a good indicator of iodine status [21]. Additionally, serum Tg concentrations showed minimal day-to-day variation compared to spot UIC samples following the introduction of a salt iodization program [52]. This lack of variation in Tg concentrations compared to UIC may suggest that Tg is more representative of long-term iodine intake in a population, especially in areas with endemic goiter because it may reflect overall thyroid cell mass [35,52]. Overall, however, there are limited studies on serum Tg as a biomarker of individual iodine status and further research is needed to establish reference concentrations in healthy, iodine-sufficient individuals and to understand the relationship between Tg and recent iodine intake.

The secondary purposes of the current study were to evaluate the association between baseline 24 h UIC and habitual iodine intake and observe the contribution of iodine-containing foods to total iodine intake assessed by an iodine-specific FFQ. Milk and total dairy intake correlated positively with total iodine intake as estimated by the iodine-specific FFQ. We did find positive associations between UIC and milk and fish/seafood intake. As with all self-reported data, there is potential for under or overreporting of nutrient intake [29] as could be the case with our iodine-specific FFQ. However, previous studies [12,54,55] have found positive correlations with UIC and milk and dairy intake, including one from our lab which reported total dairy and egg intake to predict approximately 20% variance in 24 h UIC [29]. A review by Reijden et al. estimated that milk and dairy contribute ~13–64% of recommended daily iodine intake based on country-specific food intake data [55]. Iodine content in dairy is a reliable, although variable, source of dietary iodine. Iodine enters cow’s milk either through the cow’s ingestion of water, feed, vegetation, or through exposure to iodophor disinfectants. Such disinfectants are used in the US to clean cow teats and udders, milking equipment, and other milk-holding containers and transporting trucks [56]. However, the iodine content of milk can vary greatly, from 33 to 534 μg/L according to a recent study [55]. Such variations are due to differences in agricultural practices, seasonal variations, milk yield, type, concentration, and application method of iodophor sanitizers, type of cow feed, goitrogen intake by dairy cows, and seasonal variations of pasture versus prepared feed [12,55]. Alternately, dairy and milk are easily recognized and recalled compared to other iodine sources such as iodized salt. Dairy items may be a common iodine source because they can be consumed through easily recalled sources including the habitual glass of milk or as milk added to breakfast cereals or consumed as convenient dairy items such as yogurts and cottage cheese. 

Although the current study has several strengths, there are limitations which include a smaller sample size and short study duration. The smaller sample size allowed for control and accountability in data collection methodology, but overall findings may not be generalizable to other populations with more extremes in iodine status or in those who are pregnant or lactating. Our participants, for example, were screened for normal thyroid function and most had iodine intake that met the daily US recommendation. Additionally, excess sodium consumption may have contributed to excess fluid retention which could influence serum iodine and Tg biomarker concentrations, particularly on the 3× RDA diet. A longer study duration (perhaps weeks or months) may have allowed for observation of whether serum iodine and thyroglobulin biomarkers would be sensitive to longer term changes in dietary iodine intake. Future studies from our laboratory will further evaluate the use of biomarkers of iodine status using longer titration diet periods and an iodine source other than iodized salt (to avoid risk of excess sodium consumption). Limitations apart from the study design include the high variability of iodine content in food and the absence of iodine in the USDA Food Composition Database, making it difficult to reliably assess iodine intake and make comparisons to iodine status. The iodine content of soil naturally varies and thus the iodine content of plants and animals, agriculture products, saltwater fish and seafood is highly variable. The FFQ used averages of iodine content from the TDS to estimate iodine intake in foods. However, the average values are highly variable. For example, the iodine content of salt water fish fillets ranged from 0.122 to 0.922 μg/g, while a fillet of fresh water fish ranged from 0.005 to 0.082 μg/g in a study by Eckhoff and Maage [57]. 

## 5. Conclusions

The current study provides preliminary support to suggest that 24 h UIC and serum iodine may be indicative of individual iodine status, at least over the short term (3 days) when iodine status is mostly stable. Results provide support that 24 h UIC is likely the most sensitive indicator of individual iodine status at this time and still may be the best biomarker to assess iodine status. However, serum iodine is easier to measure at a single blood draw than single or multiple 24 h UICs and would provide a convenient method to assess iodine status, especially for pregnant and lactating women. Serum Tg was not sensitive to short term changes in iodine intake, but future studies should evaluate its use as a long-term iodine status marker. This study highlights the need for additional research to identify individual iodine biomarkers. Continuing to explore these markers to promptly identify iodine deficiencies in women of reproductive age and pregnant and lactating women is especially important so that dietary interventions can be recommended, and deficiencies can be corrected.

## Figures and Tables

**Table 1:** Descriptive Characteristics and Baseline Biochemical Values of Participants by Sex.

	Mean ± SEM	
	Male	Female
Age	26.6 ± 1.1	36.0 ± 7.4
Height (cm)	183.2 ± 2.8	166.4 ± 2.6
Weight (kg)	82.8 ± 4.4	69.5 ± 11.3
BMI (kg/M^2^)	24.7 ± 1.1	24.8 ± 3.4
Body Fat (%)	20.7 ± 3.8	31.3 ± 4.5
Hemoglobin (g/dL)	16.5 ± 0.2	14.6 ± 0.3
Hematocrit (%)	49.0 ± 0.6	43.5 ± 1.1
TSH (mcIU/mL)	2.0 ± 0.3	1.5 ± 0.2
Thyroglobulin (ng/mL)	17.5 ± 5.2	16.9 ± 4.2
24 h UIC (μg/L)	126.6 ± 28.7	142.4 ± 29.6

**Table 2:** Titration Diet Effects on Markers of Iodine Status (Mean ± SEM).

Iodine Marker	Minimal Iodine	RDA	3× RDA	Normal Range
24 h UIC (μg/L)	19.1 ± 6.0	31.2 ± 9.9	173.9 ± 55.0	100–199 μg/L
24 h UIE (μg/day)	82.7 ± 8.5	174.1 ± 15.2	401.4 ± 63.8	N/A
Serum Iodine (ng/mL)	62.1 ± 3.3	61.5 ± 3.6	66.8 ± 3.8	40–92 ng/mL
Tg (ng/mL)	17.2 ± 3.2	17.1 ± 2.9	17.8 ± 3.1	≤33 ng/mL

## Data Availability

The data presented in this study are available on reasonable request from the corresponding author. The data are not publicly available due to privacy concerns.

## Appendix A

**Food Frequency Questionnaire (Ffq)** **For each food listed, check the box indicating how often on average you have used the amount specified during the last month**									
**Foods Consumed**	**Never or** **<1 per mo.**	**1–3 per** **Month**	**1 per** **Week**	**2–4 per** **Week**	**5–6 per** **Week**	**1 per** **Day**	**2–3 per** **Day**	**4–5 per** **Day**	**6+ per** **Day**
1. Milk, 1 cup									
2. Soy Milk, 1 cup									
3. Soy Protein bars, 1 bar									
4. Other non-dairy milks, 1 cup									
5. Cereal, insert yours ______________, 1 ½ cup									
6. Bread (commercial), not homemade, 1 slice									
7. Bagels, 1 each									
8. Yogurt, 1 cup									
9. Cheese, 2 oz									
10. Cottage Cheese, ½ cup									
11. Egg, 1 whole									
12. Cod, cooked, 3 ½ oz									
13. Grouper cooked, 3 ½ oz									
14. Haddock cooked, 3 ½ oz									
15. Halibut, cooked, 3 ½ oz									
16. Herring, 3 1/2 oz.									
17. Mackerel, cooked, 3 ½ oz									
18. Perch, cooked, 3½oz									
19. Sardines, canned in oil, 3 ½ oz									
20. Tukaoua, cooked, 3 ½ oz									
21. Tuna, Albacore, or light, canned, 3 ½ oz									
22. Walleye, cooked, 3 ½ oz									
23. Other fish, 3 ½ oz Insert yours _________									
24. Clams, 4 oz									
25. Crabmeat, 4 oz									
26. Lobster, 4 oz									
27. Mussels, 4 oz									
28. Oysters, 4 oz									
29. Scallops, 4 oz									
30. Shrimp, 4 oz									
31. Seaweed Snacks, insert yours _____________									
32. Seaweed Sushi,1 Roll (or 6 pieces)									
33. Iodized Table Salt									
34. Other Salt (sea salt, Pink Himalayan, Kosher)									
Multi Vitamin, 1 Tablet (Type and Brand) _________________									
3.Kelp (or other iodine supplement), 1 Tablet									
If you take any of the above, please list which you use, brand name, etc.									

## References

[1] Zimmermann M.B. (2009). Iodine Deficiency. Endocr. Rev..

[2] Markel H. (1987). When It Rains It Pours: Endemic Goiter, Iodized Salt, and David Murray Cowie, MD. Public Health Then Now.

[3] Caldwell K., Makhmudov A., Ely E., Jones R., Wang R.Y. (2011). Iodine Status of the U.S. Population, National Health and Nutrition Examination Survery, 2005–2006 and 2007–2008. Thyroid.

[4] Panth P., Guerin G., DiMarco N.M. (2019). A Review of Iodine Status of Women of Reproductive Age in the USA. Biol. Trace Elem. Res..

[5] Caldwell K.L., Pan Y., Mortensen M.E., Makhmudov A., Merrill L., Moye J. (2013). Iodine Status in Pregnant Women in the National Children’s Study and in U.S. Women (15–44 Years), National Health and Nutrition Examination Survey 2005–2010. Thyroid.

[6] Hynes K.L., Seal J.A., Otahal P., Oddy W.H., Burgess J.R. (2019). Women Remain at Risk of Iodine Deficiency during Pregnancy: The Importance of Iodine Supplementation before Conception and throughout Gestation. Nutrients.

[7] Leung A.M., LaMar A., He X., Braverman L.E., Pearce E.N. (2011). Iodine Status and Thyroid Function of Boston-Area Vegetarians and Vegans. J. Clin. Endocrinol. Metab..

[8] Groufh-Jacobsen S., Hess S.Y., Aakre I., Gjengedal E.L.F., Pettersen K.B., Henjum S. (2020). Vegans, Vegetarians and Pescatarians Are at Risk of Iodine Deficiency in Norway. Nutrients.

[9] Eveleigh E.R., Coneyworth L.J., Avery A., Welham S.J.M. (2020). Vegans, Vegetarians, and Omnivores: How Does Dietary Choice Influence Iodine Intake? A Systematic Review. Nutrients.

[10] Remer T., Neubert A., Manz F. (1999). Increased Risk of Iodine Deficiency with Vegetarian Nutrition. Br. J. Nutr..

[11] Krajcovicova-Kudlackova M., Bučková K., Klimeš I., Šeboková E. (2003). Iodine Deficiency in Vegetarians and Vegans. Ann. Nutr. Metab..

[12] Pearce E.N., Pino S., He X., Bazrafshan H.R., Lee S.L., Braverman L.E. (2004). Sources of Dietary Iodines Bread, Cows’ Milk, and Infant Formula in the Boston Area. J. Clin. Endocrinol. Metab..

[13] WHO, UNICEF, ICCIDD (2007). Assessment of Iodine Deficiency Disorders and Monitoring Their Elimination.

[14] Tayie F.A.K., Jourdan K. (2010). Hypertension, Dietary Salt Restriction, and Iodine Deficiency among Adults. Am. J. Hypertens..

[15] Fuge R., Johnson C.C. (1958). The Geochemistry of Iodine—A Review. Br. Geol. Surv..

[16] USDA (2020). Dietary Guidelines for Americans 2020–2025.

[17] Henney J.E., Taylor C.L., Boon C.S. (2010). Strategies to Reduce Sodium Intake in the United States.

[18] Appel L.J., Frohlich E.D., Hall J.E., Pearson T.A., Sacco R.L., Seals D.R., Sacks F.M., Smith S.C., Vafiadis D.K., Van Horn L.V. (2011). The Importance of Population-Wide Sodium Reduction as a Means to Prevent Cardiovascular Disease and Stroke: A Call to Action from the American Heart Association. Circulation.

[19] Hetzel B.S., Dunn J.T. (1989). The Iodine Deficiency Disorders: Their Nature and Prevention. Annu. Rev. Nutr..

[20] Haap M., Roth H.J., Huber T., Dittmann H., Wahl R. (2017). Urinary Iodine: Comparison of a Simple Method for Its Determination in Microplates with Measurement by Inductively-Coupled Plasma Mass Spectrometry. Sci. Rep..

[21] Wainwright P., Cook P. (2019). The Assessment of Iodine Status—Populations, Individuals and Limitations. Ann. Clin. Biochem..

[22] Rasmussen L.B., Ovesen L., Christiansen E. (1999). Day-to-Day and within-Day Variation in Urinary Iodine Excretion. Eur. J. Clin. Nutr..

[23] Remer T., Fonteyn N., Alexy U., Berkemeyer S. (2006). Longitudinal Examination of 24-h Urinary Iodine Excretion in Schoolchildren as a Sensitive, Hydration Status-Independent Research Tool for Studying Iodine Status. Am. J. Clin. Nutr..

[24] Manousou S., Stål M., Larsson C., Mellberg C., Lindahl B., Eggertsen R., Hulthén L., Olsson T., Ryberg M., Sandberg S. (2018). A Paleolithic-Type Diet Results in Iodine Deficiency: A 2-Year Randomized Trial in Postmenopausal Obese Women. Eur. J. Clin. Nutr..

[25] Jin X., Jiang P., Liu L., Jia Q., Liu P., Meng F., Zhang X., Guan Y., Pang Y., Lu Z. (2017). The Application of Serum Iodine in Assessing Individual Iodine Status. Clin. Endocrinol..

[26] Ma Z.F., Skeaff S.A. (2014). Thyroglobulin as a Biomarker of Iodine Deficiency: A Review. Thyroid.

[27] Solomon A. (1956). Pathways of Iodine Metabolism. Am. J. Med..

[28] Mao F., Ko Y.C., Chen M.-L. (1990). Stability of Iodine in Human Sweat. Jpn. J. Physiol..

[29] Gostas D.E., Larson-Meyer D.E., Yoder H.A., Huffman A.E., Johnson E.C. (2020). Dietary Relationship with 24-Hour Urinary Iodine Concentrations of Young Adults in the Mountainous West Region of the United States. Nutrients.

[30] Halliday T.M., Peterson N.J., Thomas J.J., Kleppinger K., Hollis B.W., Larson-Meyer D.E. (2011). Vitamin D Status Relative to Diet, Lifestyle, Injury, and Illness in College Athletes. Med. Sci. Sports Exerc..

[31] Gerow K., Bozeman B., Grossman G.D. (2021). Response Feature Analysis for Repeated Measures in Ecological Research. Bull. Ecol. Soc. Am..

[32] Zimmermann M.B. (2008). Methods to Assess Iron and Iodine Status. Br. J. Nutr..

[33] Censi S., Watutantrige-Fernando S., Groccia G., Manso J., Plebani M., Faggian D., Mion M.M., Venturini R., Andrisani A., Casaro A. (2019). The Effects of Iodine Supplementation in Pregnancy on Iodine Status, Thyroglobulin Levels and Thyroid Function Parameters: Results from a Randomized Controlled Clinical Trial in a Mild-to-Moderate Iodine Deficiency Area. Nutrients.

[34] Zimmermann M.B., De Benoist B., Corigliano S., Jooste P.L., Molinari L., Moosa K., Pretell E.A., Al-Dallal Z.S., Wei Y., Chen Z.P. (2006). Assessment of Iodine Status Using Dried Blood Spot Thyroglobulin: Development of Reference Material and Establishment of an International Reference Range in Iodine-Sufficient Children. J. Clin. Endocrinol. Metab..

[35] Reinhardt W., Luster M., Rudorff K.H., Heckmann C., Petrasch S., Lederbogen S., Haase R., Saller B., Reiners C., Reinwein D. (1998). Effect of Small Doses of Iodine on Thyroid Function in Patients with Hashimoto’s Thyroiditis Residing in an Area of Mild Iodine Deficiency. Eur. J. Endocrinol..

[36] Huda S.N., Grantham-McGregor S.M., Tomkins A. (2001). Cognitive and Motor Functions of Iodine-Deficient but Euthyroid Children in Bangladesh Do Not Benefit from Iodized Poppy Seed Oil (Lipiodol). J. Nutr..

[37] Tonglet R., Bourdoux P., Minga T., Ermans A.-M. (1992). Efficacy of Low Oral Doses of Iodized Oil in the Control of Iodine Deficiency in Zaire. N. Engl. J. Med..

[38] Phillips D.I.W., Osmond C. (1989). Iodine Supplementation with Oral or Intramuscular Iodized Oil. A Two-Year Follow-up of a Comparative Trial. Int. J. Epidemiol..

[39] Rohner F., Zimmermann M.B., Jooste P.L., Pandav C., Caldwell K., Raghavan R., Raiten D.J. (2014). Biomarkers of Nutrition for Development Iodine Review. J. Nutr..

[40] Hetzel B.S., Zimmermann M.B. (1993). The Iodine Deficiency Disorders. Iodine Defic. Eur..

[41] Andersen S., Karmisholt J., Pedersen K.M., Laurberg P. (2008). Reliability of Studies of Iodine Intake and Recommendations for Number of Samples in Groups and in Individuals. Br. J. Nutr..

[42] Soldin O.P. (2002). Controversies in Urinary Iodine Determinations. Clin. Biochem..

[43] Moreno-Reyes R., Carpentier Y.A., MacOurs P., Gulbis B., Corvilain B., Glinoer D., Goldman S. (2011). Seasons but Not Ethnicity Influence Urinary Iodine Concentrations in Belgian Adults. Eur. J. Nutr..

[44] Als C., Helbling A., Peter K., Haldimann M., Zimmerli B., Gerber H. (2000). Urinary Iodine Concentration Follows a Circadian Rhythm: A Study with 3023 Spot Urine Samples in Adults and Children. J. Clin. Endocrinol. Metab..

[45] Zou Y., Li H., Pang J., Liu X., Tian L., Yu S., Wang D., Hou L., Yin Y., Ma C. (2021). An Evaluation of Urine and Serum Iodine Status in the Population of Tibet, China: No Longer an Iodine-Deficient Region. Nutrition.

[46] Yu S., Wang D., Cheng X., Zhang Q., Wang M., Guo H., Yu B., Zhang X., Xia L., Sun D. (2020). Establishing Reference Intervals for Urine and Serum Iodine Levels: A Nationwide Multicenter Study of a Euthyroid Chinese Population. Clin. Chim. Acta.

[47] Næss S., Aakre I., Kjellevold M., Dahl L., Nerhus I., Midtbø L.K., Markhus M.W. (2019). Validation and Reproducibility of a New Iodine Specific Food Frequency Questionnaire for Assessing Iodine Intake in Norwegian Pregnant Women. Nutr. J..

[48] Brantsæter A.L., Haugen M., Julshamn K., Alexander J., Meltzer H.M. (2009). Evaluation of Urinary Iodine Excretion as a Biomarker for Intake of Milk and Dairy Products in Pregnant Women in the Norwegian Mother and Child Cohort Study (MoBa). Eur. J. Clin. Nutr..

[49] Tan L.M., Charlton K.E., Tan S.Y., Ma G., Batterham M. (2013). Validity and Reproducibility of an Iodine-Specific Food Frequency Questionnaire to Estimate Dietary Iodine Intake in Older Australians. Nutr. Diet..

[50] Michalke B., Witte H. (2015). Characterization of a Rapid and Reliable Method for Iodide Biomonitoring in Serum and Urine Based on Ion Chromatography-ICP-Mass Spectrometry. J. Trace Elem. Med. Biol..

[51] Rasmussen L.B., Ovesen L., Knudsen N., Laurberg P., Perrild H. (2002). Relations between Various Measures of Iodine Intake and Thyroid Volume, Thyroid Nodularity, and Serum Thyroglobulin. Am. J. Clin. Nutr..

[52] Vejbjerg P., Knudsen N., Perrild H., Laurberg P., Carlé A., Pedersen I.B., Rasmussen L.B., Ovesen L., Jørgensen T. (2009). Thyroglobulin as a Marker of Iodine Nutrition Status in the General Population. Eur. J. Endocrinol..

[53] Yang D., Yan Hui G., Zhuo Ying F., Fan Gang M., Li Jun F., Dian Jun S. (2017). Serum Thyroglobulin—A Sensitive Biomarker of Iodine Nutrition Status and Affected by Thyroid Abnormalities and Disease in Adult Populations. Biomed. Environ. Sci..

[54] Lee K.W., Shin D., Cho M.S., Song W.O. (2016). Food Group Intakes as Determinants of Iodine Status among US Adult Population. Nutrients.

[55] van der Reijden O.L., Zimmermann M.B., Galetti V. (2017). Iodine in Dairy Milk: Sources, Concentrations and Importance to Human Health. Best Pract. Res. Clin. Endocrinol. Metab..

[56] Pennington J.A.T. (1990). Iodine Concentrations in US Milk: Variation Due to Time, Season, and Region. J. Dairy Sci..

[57] Eckhoff K.M., Maage A. (1997). Iodine Content in Fish and Other Food Products from East Africa Analyzed by ICP-MS. J. Food Compos. Anal..

